# Draft Genome Sequences of Three O157 Enteropathogenic *Escherichia coli* Isolates

**DOI:** 10.1128/genomeA.00516-13

**Published:** 2013-07-18

**Authors:** Tracy H. Hazen, Jason W. Sahl, Claire M. Fraser, Michael S. Donnenberg, Flemming Scheutz, David A. Rasko

**Affiliations:** Department of Microbiology and Immunology, University of Maryland School of Medicine, Baltimore, Maryland, USAa; Institute for Genome Sciences, University of Maryland School of Medicine, Baltimore, Maryland, USAb; Department of Medicine, University of Maryland School of Medicine, Baltimore, Maryland, USAc; WHO Collaborating Centre for Reference and Research on *Escherichia* and *Klebsiella*, Statens Serum Institut, Copenhagen, Denmarkd

## Abstract

We report the draft genome sequences of three enteropathogenic *Escherichia coli* (EPEC) isolates that display the O157 serogroup but do not have the Shiga toxin genes (*stx*), which are characteristic of O157 enterohemorrhagic *E. coli* (EHEC). *E. coli* strain RN587/1 has the O157:H8 serotype and possesses the EAF plasmid characteristic of typical EPEC (J. B. Kaper, J. P. Nataro, and H. L. Mobley, Nat. Rev. Microbiol. 2:123–140, 2004). The other two isolates, strains C844-97 and C639-08, are both O157:H45 and possess the locus of enterocyte effacement (LEE) pathogenicity island; however, they do not contain the EAF plasmid or the *stx*-carrying phage.

## GENOME ANNOUNCEMENT

Attaching and effacing *Escherichia coli* (AEEC) is characterized by the presence of the locus of enterocyte effacement (LEE) pathogenicity island (1–3) and includes enterohemorrhagic *E. coli* (EHEC), which expresses Shiga toxins (encoded by *stx*_1_ and/or *stx*_2_), and typical enteropathogenic *E. coli* (EPEC), which expresses the EAF plasmid-carried bundle-forming pilus gene (*bfp*) (1, 4, 5). O157 EHEC has been identified as one of the most frequent agents of severe gastrointestinal illness and hemolytic-uremic syndrome (HUS) in the United States (6–8). O157:H45 EPEC isolates with the genotype *bfp*^+^ have been previously associated with an outbreak of diarrhea in Japan (9). Additionally, O157:non-H7 *E. coli* has been isolated from human, animal, and water sources (10, 11), and a recent study demonstrated that the O157-antigen gene cluster is present in *E. coli* strains with diverse genetic backgrounds rather than being restricted to O157:H7 EHEC (12), suggesting that the serogroup alone is not indicative of increased virulence.

Genomic DNA was isolated from an overnight culture using the Sigma GenElute kit (Sigma-Aldrich) and was sequenced at the University of Maryland School of Medicine, Institute for Genome Sciences, Genome Resource Center (http://www.igs.umaryland.edu/). The genome sequence of the RN587/1 isolate was generated using 8-kb-insert paired-end libraries on the 454 Titanium system (Roche), and the genome sequences of the C844-97 and C639-08 isolates were generated using paired-end libraries with 300-bp inserts on the Illumina HiSeq2000. The draft genome sequences were assembled using the Celera assembler (13) for 454 sequence data or the Velvet assembly program (14) for the Illumina data. The resulting genome sequences contained an average of 170 contigs per isolate (range, 117 to 243).

These isolates represent the first draft genome sequences of three O157:non-H7 isolates from the EPEC pathotype. Isolate RN587/1 exhibits the O157:H8 serotype and was identified as EPEC by the presence of the LEE and the *bfpA* gene (15). The other two isolates, C844-97 and C639-08, are isolates from human diarrhea and are serotyped as O157:H45. C844-97 was isolated in Japan and C639-08 was isolated in Denmark. None of these isolates described possess the Shiga toxin genes, *stx*_1_ and *stx*_2_, which are characteristic of EHEC; thus, they were identified as EPEC. In a whole-genome phylogeny, the genomes from these O157:non-H7 isolates formed a distinct group within the B2 phylogenetic lineage of all *E. coli* strains and are most similar to the EPEC1 pathotype (data not shown). Furthermore, all three of the O157 EPEC isolates have the alpha intimin type, which is characteristic of EPEC1 and other members of B2 (12). The bundle-forming pilus genes of the EAF plasmid that are characteristic of typical EPEC were identified in RN587/1 but not C844-97 or C639-08. However, the EAF plasmid was originally detected in C844-97, suggesting this isolate has recently lost the virulence plasmid.

To our knowledge, these are among the first O157:non-H7 EPEC genome sequences to be released into the public domain.

### Nucleotide sequence accession numbers.

The genome sequence data have been deposited in GenBank with accession numbers ADUS00000000, AIBZ00000000, and AIBH00000000 for isolates RN587/1, C844-97, and C639-08, respectively.
